# Influence of socio‐economic and agronomic factors on aflatoxin and fumonisin contamination of maize in western Kenya

**DOI:** 10.1002/fsn3.1070

**Published:** 2019-06-11

**Authors:** Nancy Karimi Njeru, Charles Aura Odhiambo Midega, James Wanjohi Muthomi, John Maina Wagacha, Zeyaur Rahman Khan

**Affiliations:** ^1^ International Centre of Insect Physiology and Ecology (icipe) Nairobi Kenya; ^2^ Department of Plant Science and Crop Protection University of Nairobi Nairobi Kenya; ^3^ School of Biological Sciences University of Nairobi Nairobi Kenya

**Keywords:** agronomic practices, management, Mycotoxins, push‐pull, *Zea mays*

## Abstract

Consumption of maize contaminated with mycotoxins has been associated with detrimental health effects. A farm survey covering 116 push‐pull and 139 non‐push‐pull cropping systems was conducted to determine the socio‐economic and agronomic factors that influence farmers’ knowledge on incidence and contamination of maize by ear rots and associated mycotoxins in western Kenya. All the respondents were smallholder farmers between the ages of 23 and 80 years, with 50% of them being female. Maize samples were collected from the standing crop in the field of each interviewed farmer and analyzed for aflatoxin and fumonisin. Only a small proportion of farmers had knowledge of aflatoxin and ear rots in maize. Overall, less than 20% of maize samples were contaminated with both aflatoxin and fumonisin, and more maize samples were contaminated with fumonisin as compared to aflatoxin. Proportions of maize samples containing higher than the acceptable Kenyan regulatory threshold (10 µg/kg) for aflatoxin and European Commission regulatory threshold (1,000) µg/kg for fumonisin were lower in maize samples from push‐pull cropping system. Age of farmer and county of residence were significantly and positively associated with knowledge of aflatoxin, while cropping system, county of residence, and level of education were positively associated with knowledge of maize ear rots. There was strong correlation between knowledge of maize ear rots and knowledge of aflatoxin. Levels of both aflatoxin and fumonisin were significantly and positively associated with the use of diammonium phosphate (DAP) fertilizer at planting. Aflatoxin levels were also positively associated with stemborer damage. Agronomic practices were not significantly different between push‐pull and non‐push‐pull farmers. However, use of DAP fertilizer was the most important agronomic factor since it was associated with both aflatoxin and fumonisin contamination of maize. These results imply that creating awareness is key to mitigation of ear rots and mycotoxin contamination of maize. The results also suggest that the levels of aflatoxin and fumonisin in maize in western Kenya were influenced both by pre‐harvest agronomic practices and by the cropping system adopted, push‐pull or not.

## 1 INTRODUCTION

Maize (*Zea mays* L.) is the staple food for majority of households in Kenya, with nearly all agricultural households growing the crop. Compared to other food crops, maize production occupies more land, with over 70% of its production being done by smallholder mixed farmers (Keya & Rubaihayo, 2013; Kibet, 2011). Maize is used as food for humans, as feed for livestock, and as industrial raw material for many manufactured maize‐based products. However, the quality of maize is compromised by numerous constraints, which include fungal diseases, such as ear rots and kernel infections. Ear rot is a major disease of maize worldwide that is visually characterized by moldiness of kernels at different points of the cob depending on the causative fungi during the growth of the crop, at harvest and during storage (Bigirwa, Kaaya, Sserurwa, Adipala, & O. S., 2007). Moldy kernels have low integrity compared to healthy kernels. The predominant maize ear rot causative fungi are determined by the climatic conditions of a region. Common types of maize ear rots are *Fusarium*, *Penicillium*, *Aspergillus*, *Diplodia,* and *Giberella* (www.krugerseed.com). Some ear rot fungi such as *Fusarium* and *Aspergillus* spp. pose health risks by contaminating maize with associated mycotoxins under appropriate conditions of moisture and temperature (Dragich & Nelson, 2014). Maize ear rots also reduce grain yield, since the rotten grains are removed during shelling.

Mycotoxins are toxic compounds naturally produced by certain fungi as secondary products of their biosynthetic pathway (Hendrich, 2017; WHO, 2018). When produced in food like maize and maize‐based food products, mycotoxins become a threat to food and feed safety because they are poisonous to humans and animals. Consumption of mycotoxin‐contaminated foods has been linked with adverse health effects such as exacerbation of the symptoms of diseases like HIV/AIDS and malaria, suppression of the immune system, cancers of vital organs, and even death in humans (Lewis et al., 2005; WHO, 2018; Williams, Aggarwal, Jolly, Phillips, & Wang, 2005). Humans can also be exposed to mycotoxins through inhalation or contact with contaminated foods (Paterson & Lima, 2010). In livestock, mycotoxins in feed have been associated with reduced feed intake, degradation of ruminal microflora, reduced productivity, and intoxication (Fink‐Gremmels, 2008). Aflatoxin, fumonisin, deoxynivalenol, and zearalenone are common mycotoxins of maize (Broggi et al., 2007).

Mycotoxin contamination of maize is usually influenced by factors such as climatic conditions during the cropping season, production practices, and physical factors, mainly temperature and moisture. However, drivers for contamination are different for different mycotoxins. Warm temperature and erratic weather patterns encourage infection of maize grains by toxigenic fungi such as *A. flavus* (Cotty & Jaime‐Garcia, 2007). A previous report indicated that proper crop management practices effectively controls *A. flavus* and *F. verticillioides* and associated mycotoxins (Bruns, 2003). Tillage practices, handling of maize stovers after harvest, host susceptibility, use of fertilizers, rate of maturity of cultivar, kernel breakage, shape of kernel, presence of rotten kernels at harvest, and continuous cultivation also influence proliferation of some ear rot fungi (Mutiga et al., 2017, 2014). For instance, farmer practice of handling maize stovers after harvest could influence population of ear rot fungi and associated mycotoxins, since most mycotoxigenic fungi survive as saprophytes on crop residues (Chulze et al., 2015; Mutiga et al., 2017). Drying duration after harvest for the maize also significantly influences production of aflatoxin because of *A. flavus* infection at specific moisture and humidity level (Mbuge et al., 2016). Edwards (2004) reported that exposure to mycotoxins can also be due to insect damage to maize cobs. Feeding insects can either act as vector of fungal pathogens by transferring the fungi or expose the cob to infection from the atmosphere. Insect damage is of most important concern for *Fusarium* spp. mycotoxins such as fumonisin (Sobek & Munkvold, 1999).

A recent study reported reduced incidence and severity of maize ear rots and associated mycotoxins with the push‐pull technology (Owuor, Midega, Obonyo, & Khan, 2018). However, cropping systems and management practices as drivers of mycotoxin contaminations, including underlying mechanisms, have remained unstudied in the region. Push‐pull is a farming system that involves intercropping cereals with a “push” crop and a border “pull” crop around the plot (Khan, Pickett, Berg, Wadhams, & Woodcock, 2000). In maize farming, the system has three components: the maize, *Desmodium* spp. (commonly known as desmodium) as the intercrop, and *Brachiaria* or Napier grass (*Pennisetum purpureum*) as the border crop. Desmodium roots produce allelopathic chemicals which induce suicidal germination of striga seeds, thus suppressing the development of the weed (Khan, Hassanali, Pickett, Wadhams, & Muyekho, 2003). Desmodium also improves soil nutrition through biological nitrogen fixation and phosphorus availability (Khan et al., 2000). Desmodium foliage emits semiochemicals that repel gravid stemborer moths (Midega, Jonsson, Khan, & Ekbom, 2014; Midega, Pittchar, Pickett, Hailu, & Khan, 2018), which are simultaneously attracted to the “pull” plants where they lay their eggs. The border crop, however, has characteristics that cause high mortality of the larvae (Khan et al., 2000). The objective of this study was to establish socio‐economic and agronomic factors aggravating aflatoxin and fumonisin contamination of maize in western Kenya, as well as compare the practices of push‐pull and non‐push‐pull farmers. Farmers’ knowledge of the two mycotoxins and maize ear rots and the association of their knowledge with farmers’ socio‐economic status were also determined.

## 2 MATERIALS AND METHODS

### 2.1 Description of the Study sites

The study was conducted in selected sub‐counties in five counties of western Kenya: Kisumu, Vihiga, Siaya, Kakamega, and Migori. These five counties represent counties where smallholder maize farmers have widely adopted the push‐pull cropping system for stemborer and striga management. In the five counties, maize is the main food crop and source of income. The agro‐ecology parameters of the counties are shown in Table 1. A total of 255 farmers were randomly selected from a list provided by the International Centre of Insect Physiology and Ecology (*icipe*) field staff located in the counties; 116 of these were farmers who had adopted the push‐pull technology, while 139 had not (had plots planted to maize monocrop or intercropped with a food legume).

**Table 1:** Agro‐ecological characteristics of subcounties in Migori, Vihiga, Siaya, Kakamega and Kisumu counties where maize samples were collected

County	Subcounties	AEZ	Altitude (m asl)	Rainfall^a^	Temperature^b^
Siaya	Ugunja	LM 1, 2	1,200–1,500	1,450–1,900	20.9–22.3
Kakamega	Khwisero	UM 1, LM1	1,300–1,900	1,650–>2,000	18.5–22.2
Kisumu	Kisumu west	LM 2, 3	1,140–1,500	1,050–1,600	20.9–22.7
Migori	Rongo, Awendo	LM 1, 2	1,300–1550	1,300–1,800	20.4–21.7
Vihiga	Emuhaya, Luanda	UM 1, LM1	1,300–1,900	1,650–>2,000	−22.2

Abbreviations: asl, above sea level; AEZ, agro‐ecological zones; LM, lower midland; UM, upper midland.

Source: Jaetzold, Schmidt, Hornetz, and Shisanya (2009), Jaetzold et al. (2010).

a Average annual rainfall (mm).

b Average annual temperature (°C).

### 2.2 Field survey and collection of maize samples

A household survey was conducted using a pre‐tested semi‐structured questionnaire between January and February 2017. Face‐to‐face interviews were conducted by trained enumerators to obtain information on socio‐economic factors and agronomic practices of maize farmers that influence understanding of and be associated with aflatoxin and fumonisin contamination of maize, respectively. Socio‐economic data collected included age of the farmer, sex of the farmer, level of education, acreage under maize production, membership of welfare group(s), experience in maize farming, knowledge of aflatoxin and fumonisin, source of funding for farm inputs, and average annual family income. Agronomic data collected included cropping system (push‐pull or otherwise), varieties of maize grown, sources of seeds, any crop rotation program, intercropping practice, use of fertilizers, tillage method, use of maize stovers after harvest, knowledge of maize ear rots, and causes and management of maize ear rots. The questionnaire was set in English, and the enumerators interpreted the questions to farmers in local languages of the study area. Ten to 20 maize cobs, depending on the size of cob, were collected from standing crop from farms of each respondent. The cobs were sun‐dried, manually shelled and finely ground (Bunn‐O‐Matic Corporation Coffee Mill, G3‐000) before storage at 4°C until analyses.

### 2.3 Detection and quantification of aflatoxin levels in maize

Twenty grams sub‐samples were weighed in duplicate for each sample and extracted with 100 ml of 70% methanol. The samples were mixed by shaking in sealed containers for 2 min. The particulate matter was allowed to settle; the extracts filtered through Whatman no.1 filter paper and the filtrate collected for testing. Aflatoxin levels were quantified by direct competitive enzyme‐linked immunosorbent assay (ELISA) (Helica Biosystems Inc, Santa Ana, USA) following manufacturer's instructions. The lower and the upper limits of detection for the aflatoxin test kit were 1 and 20 µg/kg, respectively. A calibration curve for the aflatoxin standards was plotted and used to compare the optical densities of the samples with those of the standards. Samples with aflatoxin levels above the upper limit of detection were diluted and the toxin levels quantified again.

### 2.4 Detection and quantification of fumonisin levels in maize

Twenty grams sub‐samples were weighed in duplicate for each sample and extracted with 40 ml of 90% methanol. The samples were mixed by shaking in sealed containers for 1 min, after which the particulate matter was allowed to settle. The extracts were filtered through Whatman no. 1 filter paper and the filtrate collected for testing. The sample extracts were further diluted with distilled water in the ratio of 1:20. Fumonisin levels were quantified by direct competitive enzyme‐linked immunosorbent assay (ELISA) (Helica Biosystems Inc) following manufacturer's instructions. The lower and the upper limits of detection for fumonisin test kits were 100 and 6,000 µg/kg, respectively. A calibration curve for the fumonisin standards was plotted and used to compare the optical densities of the samples with those of the standards. Samples with fumonisin levels above the upper limit of detection were diluted, and the toxin levels quantified again.

### 2.5 Statistical data analyses

The survey data were analyzed using SPSS version 22 (IBM Corp, 2013). Percentages were used for basic description of socio‐economic characteristics and agronomic practices of the farmers. Aflatoxin and fumonisin data were categorized into proportion of maize samples with toxin levels falling into threshold set by Kenya Bureau of Standards (KEBS) and European Commission (EC) using the cross‐tabulation procedure in SPSS. The association between the farmers’ socio‐economic characteristics and knowledge of aflatoxin and maize ear rots was established using binary logistic regression model. Ordinal logistic regression was performed to establish the association between the farmers’ agronomic practices and aflatoxin and fumonisin levels in maize collected during the field survey.

## 3 RESULTS

### 3.1 Socio‐economic characteristics of maize farmers in five counties in western Kenya

All the respondents were smallholder farmers with low average annual income and dependent on farm produce for own consumption and sale for financing farm operations as well as supporting family financial needs (Table 2). Respondents varied in age, between 23 and 80 years. 50% of the respondents were female, between the age of 31 and 60 years. The proportion of female respondents was, however, higher (70%) for non‐push‐pull as compared to push‐pull (57%) respondents. The number of push‐pull respondents below the age of 30 years was significantly lower (*p* < 0.05) than non‐push‐push respondents. Over 80% of the respondents belonged to one or more welfare groups. As shown in Table 2, slightly over 60% of respondents had completed primary school education, indicating some level of literacy among the farmers. However, significantly less (*p* < 0.05) push‐pull respondents lacked formal education compared to non‐push‐pull respondents. Only 26.5% of the farmers had knowledge on aflatoxin, with only one farmer having heard of fumonisin. However, no farmer had knowledge of any management practices of the two mycotoxins. Most respondents had experience in maize farming of between 10 and 20 years.

**Table 2:** Socioeconomic characteristics of push‐pull and non‐push‐pull maize farmers in five counties in western Kenya

Socioeconomic trait	Proportion of farmers (%)	
	Push‐pull	Non‐push‐pull
Sex		
Female	30.0	70.0
Male	43.0	57.0
Age (years)		
18–30	4.0	11.0
31–45	33.0	37.0
46–60	38.0	33.0
Over 60	25.0	19.0
Level of education		
No formal education	2.0	5.0
Not completed primary	19.0	24.0
Completed primary school	28.0	32.0
Secondary	37.0	32.0
Tertiary	14.0	7.0
Membership to welfare group	79.0	94.0
Farming experience (years)		
<10	41.0	30.0
10–20	28.0	41.0
>20	31.0	29.0
Source of funds to purchase farm inputs		
Sale of farm produce	71.0	66.0
Casual labor	7.0	12.0
Small‐scale business	11.0	9.0
Welfare groups	15.0	11.0
Annual income (Kenyan shillings)		
20,000–35,000	38.0	41.0
36,000–55,000	18.0	19.0
56,000–75,000	15.0	11.0
76,000–100,000	13.0	18.0
Above 100,000	16.0	11.0
Knowledge on aflatoxin (yes)	32.0	22.0

Note

100 Kenyan shillings = 1 USD.

### 3.2 Agronomic practices in maize production

Ninety‐nine percent of the respondents spent the period between cropping seasons clearing and digging the fields in preparation for the subsequent season. In addition, some farmers applied compost and farmyard manure (<10%), while others grew short duration crops such as vegetables and sweet potatoes (≈15%). Tillage was mainly by use of hand hoe with significantly higher proportion of push‐pull farmers using the tool (Table 3). Most of the respondents grew maize continually, without rotating with other crops. However, for the farmers who practiced crop rotation, the key crops grown included sweet potatoes, millet, cassava, and groundnuts. The proportion of farmers intercropping maize with other food crops, mainly beans, was significantly higher (*p* < 0.05) under non‐push‐pull cropping system.

**Table 3:** Proportion (%) of push‐pull and non‐push‐pull farmers practicing various agronomic practices in western Kenya

Agronomic practice	Push‐pull	Non‐push‐pull
Tillage method		
Oxen plowing	29.0	41.0
Hand hoe digging	86.0	69.0
Crop rotation	25.0	30.0
Intercropping	60.0	85.0
Beans	53.0	71.0
Groundnuts	22.0	30.0
Others	5.0	13.0
Soil amendments		
DAP	40.0	46.0
CAN	29.0	25.0
Compost manure	44.0	22.0
Farmyard manure	64.0	75.0
Others	7.0	4.0
Maize variety		
Local	53.0	65.0
Hybrid	40.0	29.0
Method of harvesting		
Dehusking in the field	49.0	51.0
Cut stovers with cobs	43.0	40.0
Other	12.0	9.0
Use of maize stovers		
Harvest for hay	27.0	27.0
Direct grazing of cattle	34.0	32.0
Plowing in	33.0	42.0
Others	25.0	11.0
Reasons for sorting		
Avoid eating rotten maize	65.0	68.0
Keep the best for seeds	8.0	11.0
Avoid cross‐contamination	9.00	9.0
Others	16.0	12.0
Knowledge of ear rots	56.9	60.6
Use of rotten maize		
Feed livestock	70.0	64.0
Sell to local brewers	7.0	12.0
Make compost manure	2.0	6.0
Dispose	17.0	17.0
Control of ear rots		
Early harvesting	12.1	15.3
Early planting	0.0	4.4
Sorting	3.4	2.2
Other	2.6	5.1
None	40.5	35.0

Abbreviations: DAP, diammonium phosphate; CAN, calcium ammonium nitrate.

Local maize varieties were the most planted by most push‐pull and non‐push‐pull farmers (Table 3). Pioneer and DK8031 were the most common hybrid varieties planted by farmers across the counties. Other hybrid varieties grown included WH505, H513, H517, DH04, G30, H813, H113, H511, simba 61, H515, East African breed, IR, prestige, Tarco, and H516. At planting, 98% farmers amended soil with different types of organic and inorganic fertilizers (Table 3). Approximately 40% of the farmers applied diammonium phosphate (DAP) fertilizer and a significant proportion of both push‐pull and non‐push‐pull farmers applied farmyard and compost manure, although a higher proportion of push‐pull farmers used compost manure as compared to non‐push‐pull farmers.

The two most common practices of harvesting maize by the respondents were (a) dehusking maize cobs in the field, drying, and then manual shelling and (b) cutting stovers with cobs, stoking for drying, dehusking, and then manual shelling (Table 3). Upon harvesting, most respondents either harvested the maize stovers for hay or left the stovers in the farm and plowed in during cultivation. The proportion of push‐pull respondents that plowed in maize stovers was significantly lower (*p* < 0.05) compared to non‐push‐pull respondents. Other ways of handling maize stovers after harvesting included direct grazing of cattle, burning in the field and use as firewood. The harvested maize was mainly stored as shelled maize grains in polythene sacks on raised floors in the house by over 80% of respondents. A small proportion (<10%) of farmers stored maize grains in sacks directly on house floor.

Over 50% of respondents mentioned that they encountered rotten cobs, however, in low incidence and severity. About 95% of respondents hand‐sorted rotten and unwanted cobs before shelling the maize. The rotten and unwanted grains were mainly fed to livestock—cattle, poultry, and pigs (Table 3). Some respondents also used rotten maize grains for local brewing, while others mixed with clean ones for cooking or milling. The respondents estimated that grain yields were significantly higher (*p* < 0.05) in push‐pull than in non‐push‐pull cropping system, across the counties.

### 3.3 Prevalence of aflatoxin and fumonisin in maize

Overall, <20% of maize samples were contaminated with both aflatoxin and fumonisin, with proportion of maize samples without aflatoxin contamination being significantly higher (*p* < 0.05) from push‐pull farms (Table 4). Across the counties, there were less than 10% of push‐pull maize samples contaminated with aflatoxin, except in Siaya where the proportion was 12%. The proportion of non‐push‐pull maize samples contaminated with aflatoxin across the counties ranged from 6% in samples from Vihiga to 25% in samples from Siaya. All the push‐pull samples from the five counties had aflatoxin levels below the Kenya Bureau of Standards (KEBS) recommended level (10 µg/kg), while a proportion of non‐push‐pull samples from Kakamega, Siaya, and Vihiga had aflatoxin levels above the limit.

**Table 4:** Aflatoxin levels (µg/kg) in maize samples under push‐pull and non‐push‐pull cropping systems in five counties in western Kenya

Cropping system	Sample size (*n*)	Proportion of samples (%)				Highest level (µg/kg)
		<LOD	≤4	>4–≤10	>10	
Push‐pull	116	92.2	5.2	2.6	0.0	
Kakamega	18	94.4	0.0	5.6	0.0	6.4
Kisumu	21	95.2	0.0	4.8	0.0	4.5
Migori	34	91.2	5.9	2.9	0.0	6.2
Siaya	27	88.9	11.1	0.0	0.0	1.2
Vihiga	16	93.8	6.3	0.0	0.0	<LOD
Non‐push‐pull	139	88.5	7.2	0.0	4.3	
Kakamega	29	79.3	10.3	0.0	10.3	242.3
Kisumu	34	97.1	2.9	0.0	0.0	1.0
Migori	32	96.9	3.1	0.0	0.0	1.6
Siaya	28	75.0	17.9	0.0	7.1	164.9
Vihiga	16	93.8	0.0	0.0	6.3	39.8

Abbreviation: LOD, lower limit of detection.

Conversely, there were relatively higher proportions of maize samples from both push‐pull (17.2%) and non‐push‐pull (21.6%) cropping systems that were contaminated with fumonisin (Table 5). Across the counties, the proportions of push‐pull samples contaminated with fumonisin varied between 5.6% and 23.8% in samples from Kakamega and Kisumu, respectively. The proportion of non‐push‐pull maize samples contaminated with fumonisin ranged from 11.3% and 37.5% in samples from Kakamega and Vihiga, respectively. A higher proportion of non‐push‐pull samples had fumonisin levels above the European Commission (EC) recommended level (1,000 µg/kg) compared to push‐pull samples across the counties.

**Table 5:** Fumonisin levels (µg/kg) in maize samples under push‐pull and non‐push‐pull cropping systems in five counties in western Kenya

Cropping system	Sample size (*n*)	Proportion of samples (%)			Highest level (µg/kg)
		<LOD	≤1,000	>1,000	
Push‐pull	116	82.8	12.1	5.1	
Kakamega	18	94.4	5.6	0.0	210.0
Kisumu	21	76.2	19.0	4.8	1,439.3
Migori	34	79.4	14.7	5.9	4,471.3
Siaya	27	88.9	7.4	3.7	1,337.2
Vihiga	16	87.5	12.5	0.0	145.3
Non‐push‐pull	139	78.4	12.2	9.4	
Kakamega	29	89.7	3.4	6.9	10,412.3
Kisumu	34	73.5	23.5	2.9	2,325.0
Migori	32	81.3	0.0	18.8	50,769.2
Siaya	28	71.4	14.3	14.3	9,925.3
Vihiga	16	62.5	25.0	12.5	5,177.4

Abbreviation: LOD, lower limit of detection.

### 3.4 Relationship between socio‐economic characteristics of farmers and knowledge on aflatoxin and maize ear rots

Farmers’ knowledge on aflatoxin increased with increase in age, irrespective of whether the farmer practiced push‐pull or non‐push‐pull cropping system (Table 6). Specifically, the respondents between the ages of 46 and 60 years constituted 48% of the proportion of farmers with knowledge on aflatoxin and were significantly more (*p* < 0.05) knowledgeable about aflatoxin as opposed to younger age groups. The farmers from Kakamega, Migori, and Siaya were the least knowledgeable on aflatoxin compared to farmers from Vihiga. Push‐pull farmers were 0.34 times significantly less (*p* < 0.05) knowledgeable of maize ear rots compared to non‐push‐pull farmers. The average proportion of push‐pull respondents knowledgeable on ear rots was 4% lower than proportion of non‐push‐pull respondents. The farmers’ knowledge of maize ear rot was approximately 5 times higher in farmers who had primary education compared to farmers who had tertiary education. In fact, respondents who completed primary school education constituted 65% of the total number of respondents knowledgeable on ear rots. Like knowledge on aflatoxin, farmers from Kakamega, Migori, and Siaya were the least knowledgeable on ear rots. Farmers’ knowledge on aflatoxin and maize ear rots had a significant positive correlation (*r* = 0.338, *n* = 253, *p* = 0.01).

**Table 6:** Association between knowledge on aflatoxin and ear rots and socio‐economic characteristics of push‐pull and non‐push‐pull farmers in five counties in western Kenya

Socioeconomic trait	Knowledge of aflatoxin		Knowledge of maize ear rots	
	Odds ratio (95% CI)	*p* value	Odds ratio (95% CI)	*p* value
Age group (years)		0.004**		0.195
18–30	0.473 (0.03–6.45)	0.574	0.38 (0.06–2.39)	0.304
31–45	2.37 (0.65–8.71)	0.192	1.33 (0.38–4.65)	0.653
45–60	7.52 (2.14–6.43)	0.002**	2.16 (0.67–6.97)	0.196
Above 60	0^a^		0^a^	
Level of education		0.589		0.134
No formal education	0.67 (0.05–8.67)	0.760	1.38 (0.10–17.71)	0.804
Not completed primary	0.24 (0.04–1.53)	0.132	6.80 (1.19–38.56)	0.030*
Completed primary	0.37 (0.07–2.10)	0.263	5.32 (1.00–28.18)	0.050*
Secondary	0.51 (0.11–2.49)	0.408	2.59 (0.55–12.22)	0.230
Tertiary	0^a^		0^a^	
Maize farming experience (years)		0.579		0.214
Less than 10	1.07 (0.35–3.23)	0.912	2.37 (0.74–7.55)	0.145
10–20	1.67 (0.59–4.74)	0.337	0.92 (0.32–2.57)	0.873
Over 20	0^a^		0^a^	
Cropping system (push‐pull)	1.95 (0.81–4.70)	0.137	0.34 (0.13–0.82)	0.017*
Cropping system (non‐push‐pull)	0^a^		0^a^	
County		0.000***		0.000***
Siaya	0.23 (0.07–0.77)	0.016*	0.20 (0.04–0.99)	0.049*
Kisumu	1.10 (0.33–3.61)	0.878	0.49 (0.09–2.61)	0.400
Kakamega	0.00 (0.00)	0.997	0.00 (0.00)	0.996
Migori	0.04 (0.01–0.15)	0.000***	0.04 (0.00–0.19)	0.000***
Vihiga	0^a^		0^a^	

a Parameter used as reference.

* Significant at 0.05.

** Significant at 0.001.

*** Significant at 0.0001.

### 3.5 Association between agronomic practices of farmers and levels of aflatoxin and fumonisin

The levels of aflatoxin in maize samples were 3.9 times higher than 10 µg/kg in farms where DAP fertilizer was applied at planting (*p* < 0.05; Table 7). Likewise, aflatoxin levels were higher in maize sampled from farms infested by stemborer (2 times) and in maize intercropped with food crops such as sorghum and cassava (0.3 times; *p* < 0.05). Use of hand hoe tillage, compost manure, intercropping maize with beans, harvesting maize stovers as hay for cattle, plowing in maize stovers in subsequent season, and directly grazing livestock on maize stovers in the field after harvest increased the odds of maize contaminated with high levels of aflatoxin. Fumonisin levels were 0.3 times higher than 1,000 µg/kg in maize planted with DAP fertilizer (*p* < 0.05). High levels of fumonisin in maize samples were also to some extent positively influenced by most of the agricultural practices shown in Table 7.

**Table 7:** Association between levels of aflatoxin and fumonisin and agronomic practices of farmers in five counties in western Kenya

Agronomic practice	Aflatoxin		Fumonisin	
	Odds ratio (95% CI)	*p* value	Odds ratio (95% CI)	*p* value
Use DAP at planting (1 = Yes, 0 = No)	3.88 (1.22–12.38)	0.022**	0.28 (0.09–0.90)	0.032**
Use FYM at planting (1 = Yes, 0 = No)	1.07 (0.38–3.05)	0.900	0.91 (0.31–2.63)	0.860
Use compost manure at planting (1 = Yes, 0 = No)	0.61 (0.24 –1.52)	0.287	0.52 (0.21–1.29)	0.158
Hand hoe digging cultivation (1 = Yes, 0 = No)	1.12 (0.43–2.95)	0.814	1.33 (0.53–3.37)	0.541
Oxen plowing cultivation (1 = Yes, 0 = No)	0.90 (0.36–2.23)	0.817	0.88 (0.37–2.11)	0.776
Keep seeds from previous crop (1 = Yes, 0 = No)	2.01 (0.35–11.57)	0.433	1.36 (0.28–6.51)	0.704
Plant certified seeds (1 = Yes, 0 = No)	1.39 (0.23–8.37)	0.717	2.18 (0.43–10.94)	0.345
Maize variety planted (1 = Local, 0 = Hybrid)	1.14 (0.54–2.39)	0.733	1.55 (0.75–3.19)	0.239
Practice crop rotation (1 = Yes, 0 = No)	0.79 (0.42–1.49)	0.471	0.64 (0.34–1.19)	0.156
Intercrop maize with other food crops (1 = Yes, 0 = No)	0.26 (0.08–0.89)	0.032**	0.72 (0.20–2.53)	0.603
Intercrop maize with beans (1 = Yes, 0 = No)	2.64 (0.91–7.67)	0.074*	2.81 (0.91–8.68)	0.072*
Intercrop maize with groundnuts (1 = Yes, 0 = No)	0.74 (0.33–1.63)	0.452	1.46 (0.67–3.17)	0.339
Harvest for maize stovers for hay (1 = Yes, 0 = No)	0.87 (0.39–1.94)	0.736	1.03 (0.47–2.27)	0.943
Directly graze livestock on maize stovers (1 = Yes, 0 = No)	0.67 (0.33–1.39)	0.283	1.41 (0.69–2.87)	0.347
Plowing in maize stovers in the soil (1 = Yes, 0 = No)	0.74 (0.39–1.41)	0.362	1.53 (0.80–2.90)	0.196
Stemborers are the main insect (1 = Yes, 0 = No)	1.99 (1.03–3.87)	0.041**	1.05 (0.55–2.01)	0.873
Cropping system (1 = Push‐pull, 0 = Non‐push‐pull)	1.25 (0.64–2.44)	0.514	0.91 (0.48–1.70)	0.755

Note

Aflatoxin category > 10 µg/kg category was used a reference; fumonisin category > 1,000 µg/kg category was used a reference.

* Significant at 0.1.

** Significant at 0.05.

*** Significant at 0.001.

## 4 DISCUSSION

Prevalence of mycotoxins, especially aflatoxin and fumonisin in western Kenya, has been reported in a number of studies (Mutiga, Hoffmann, Harvey, Milgroom, & Nelson, 2015; Mutiga et al., 2014; Owuor et al., 2018). Most of these studies targeted stored maize and, therefore, provided no premise for developing mitigation strategies, especially preharvest. A recent study, however, reported low incidence of ear rots and levels of mycotoxins in maize grown under the push‐pull system (Owuor et al., 2018). The current study adds to this body of accumulating knowledge on mycotoxins in western Kenya by providing (a) an elucidation of the socio‐economic factors that influence the knowledge and understanding of ear rots and mycotoxins and (b) agronomic factors that aggravate contamination of maize pre‐harvest by mycotoxins within the cropping systems in the region.

The results of the survey suggest that women were the main managers of farming activities in western Kenya, which concurs with the findings of previous studies (Midega, Murage, Pittchar, & Khan, 2016; Sofa & Doss, 2011). Adults between the ages of 31 and 60 years constituted the largest age group involved in small holder maize farming in the region. The results suggested that a great proportion of the farming population in the region though fairly literate have no knowledge of maize ear rots and mycotoxins, particularly aflatoxin and fumonisin. This knowledge gap stands as a great threat to acquisition and utilization of safe food for humans and feed for animals. The agricultural practices commonly listed by the respondents included minimum tillage by hand hoe, lack of crop rotation, feeding of maize stovers to cattle, planting seeds kept from the previous crop, feeding rotten maize to livestock, and wrong reasons for sorting maize. These practices have been reported in previous studies as being incompatible with integrated management approaches for ear rots and mycotoxin contamination since they keep the maize stovers from previous cropping season longer in the farm, thus acting as a source of primary inocula of toxigenic fungi (Govaerts et al., 2008; Njeru, Muthomi, Mutegi, & Wagacha, 2016; Nyangi, 2016). When livestock are grazed on the stovers directly in the farm, they spread fungal‐infected stovers and soil from one spot of the farm to another and across neighboring farms. Therefore, the practice of handling maize stovers after harvest is therefore important in mycotoxin mitigation. For system fungi like *F*. *verticillioides*, planting infected seeds, even asymptomatically, could act as a source of secondary inocula (Parsons & Munkvold, 2012). Agricultural practices that remove, burry, or destroy infected maize stovers are likely to minimize the amount of inocula of saprophytic fungi (Edwards, 2004). Ndemera, Nyanga, Saeger, Boevre, and Landschoot (2018) reported that choice of seeds for planting significantly affects the levels of fumonisin B1 in maize.

Majority of respondents applied different types of soil amendments, both organic and mineral. This is a good practice to enhance productivity. However, the amounts applied, time of application, and status of soil fertility are not properly taken into consideration. Previous studies have reported that insufficient or excessive application of fertilizers in soil may end up enhancing contamination of maize with different mycotoxins such as fumonisin, aflatoxin, and ochratoxin (Arino et al., 2009; Blandino, Reyneri, & Vanara, 2008; Hassegawa et al., 2008). Even in this study, application of DAP fertilizer at planting had significant association with the levels of aflatoxin and fumonisin. The effect of fertilizers on levels of mycotoxins is either by alteration of the decomposition rate of crop residues or creation of physiological stress on the crop or change of canopy structure of the crop. Physiological stress could expose the maize crop to infection by opportunistic fungal pathogens such as *A. flavus*, the main producer of aflatoxin.

Farmers sorted out rotten maize by hand before shelling, a practice that reduces the levels of mycotoxins like fumonisin (Afolabi, Bandyopaghyay, Leslie, & Ekpo, 2006). However, according to the results of the survey, the rotten maize still ends up in the food chain for majority of the respondents because they mainly use it as animal feed or sell to local brewers. Feeding livestock on rotten maize and fungi‐infected maize stovers increases chronic exposure of humans to mycotoxins through animal products such as milk and eggs (Fink‐Gremmels, 2008; Jovaišienė, Bakutis, Baliukonienė, & Gerulis, 2016). Maize farming technologies that reduce incidence of maize ear rots would, therefore, reduce the levels of mycotoxins in maize as rotten maize has higher levels of mycotoxin than clean maize (Alakonya, Monda, & Ajanga, 2009).

Our findings suggest that fumonisin was a more economically important mycotoxin of maize in western Kenya during the study period as only a small proportion of maize samples was contaminated with aflatoxin, while a higher proportion was contaminated with fumonisin. Even so, a higher proportion of non‐push‐pull samples had aflatoxin and fumonisin levels above 10 µg/kg and 1,000 µg/kg, the limits set Kenya Bureau of standards (KEBS) and European Commission (EC), respectively. The proportion of maize samples with aflatoxin and fumonisin levels above the regulatory threshold was, however, lower in this study compared to some previous studies (Mutiga et al., 2015; Sirma, 2016). This was possible because in this study, samples were collected from standing crop while in the previous studies the samples were collected from storage. Maize grains contaminated with mycotoxin‐producing fungi if not properly stored would have higher levels of associated mycotoxins than they would at harvest. The amount of mycotoxins would also have been influenced by the climatic conditions of temperature and rainfall patterns during the cropping season during which the samples were collected (Tirado, Clarke, Jaykus, McQuatters‐Gollop, & Frank, 2010; Viegas, Meneses, & Viegas, 2016).

Older farmers were significantly more likely to know about aflatoxin than younger farmers. This could possibly be because the older farmers may have learnt about aflatoxin in the national news through local media during the 2004–2005 and previous aflatoxin outbreaks in lower eastern Kenya (Lewis et al., 2005). This could also be supported by the fact that knowledge of aflatoxin was not associated with the level of education. Unlike many of agronomic practices by the respondents, knowledge of existence of maize ear rots was significantly influenced by the farmers` cropping system, push‐pull or non‐push‐pull, and push‐pull farmers were less knowledgeable about maize ear rots at harvest compared to non‐push‐pull farmers. Additionally, farmers with education levels lower than the secondary level were less knowledgeable on maize ear rots as compared to those with tertiary education. The association between highest levels of education and knowledge of maize ear rot implies that literacy is key to management of maize ear rots.

Although socio‐economic and agronomic practices influenced the levels of aflatoxin and fumonisin, the maize samples from push‐pull fields had lower levels of the two mycotoxins. Push‐pull cropping system integrates insect pest management, striga control, and improvement of soil nutrition (Khan et al., 2000; Khan, Pittchar, Midega, & Pickett, 2018). As a result, the companion cropping system results in increased maize grain yield. Insect larvae infesting maize cobs are known to act as vectors for mycotoxigenic fungi such as *F. verticillioides* (Sobek & Munkvold, 1999), and therefore, it is possible that through insect control functionality, the push‐pull cropping system could contribute to reduction in levels of mycotoxins in maize.

## 5 CONCLUSIONS

From the study, we conclude that socio‐economic and agricultural factors influence mycotoxin contamination of maize in western Kenya. Indeed, some of the agronomic practices by the farmers were significantly associated with aflatoxin and fumonisin levels in the maize samples. Most of the other practices which were not significantly associated with the levels of the two toxins had reasonable (95%) confidence interval, suggesting that the practices were also important. Farmers, therefore, can play an important role in management of mycotoxins through manipulations of such practices. There is, therefore, a need to invest in mycotoxin awareness training to sensitize the farmers on good agricultural practices and management of mycotoxin.

Despite the predisposing farming practices to mycotoxin contamination of maize by both push‐pull and non‐push‐pull farmers, there were still lower levels of aflatoxin and fumonisin in maize from push‐pull cropping system. This suggests presence of several mechanisms that suppress toxigenic fungi and associated mycotoxins under push‐pull cropping system. Possibly, the less tendency of push‐pull farmers to plow‐in maize stovers after harvest could have contributed to the lower levels of the two mycotoxins. Further studies are recommended to evaluate push‐pull technology for mechanisms of management of mycotoxigenic fungi and associated mycotoxins in maize. This will inform on necessity for implementation of the cropping system as part of integrated management strategy for mycotoxin control in the region. Additionally, mycotoxin surveillance will be necessary in order to avoid future acute mycotoxicosis and help in development of robust integrated mycotoxin management tools.

## CONFLICT OF INTEREST

The authors declare that they do not have any conflict of interest.

## ETHICAL APPROVAL

This study does not involve any human or animal testing.
